# Fatal Disseminated Strongyloidiasis with Periumbilical Purpura

**DOI:** 10.4269/ajtmh.21-0464

**Published:** 2021-06-28

**Authors:** Guilherme Augusto Pivoto João, Italo Felipe Alves Antunes, Luciana Mendes dos Santos

**Affiliations:** Departamento de Ensino e Pesquisa, Fundação de Medicina Tropical Dr. Heitor Vieira Dourado, Manaus, Amazonas, Brazil

A 42-year-old male patient living with HIV in northern Brazil presented to the emergency room with a 1-week history of dyspnea and generalized itching. He had abandoned antiretroviral therapy for 2 years.

Physical examination revealed respiratory distress and hypotension. Laboratory findings included leukopenia without eosinophilia, a CD4 T-helper cell count of 7 cells/µL, and an HIV viral load of 39,331,629 copies. Chest computed tomography showed diffuse ground-glass opacities. Respiratory tract polymerase chain reaction for severe acute respiratory syndrome coronavirus 2 and *Mycobacterium tuberculosis* were negative.

**Figure 1.**
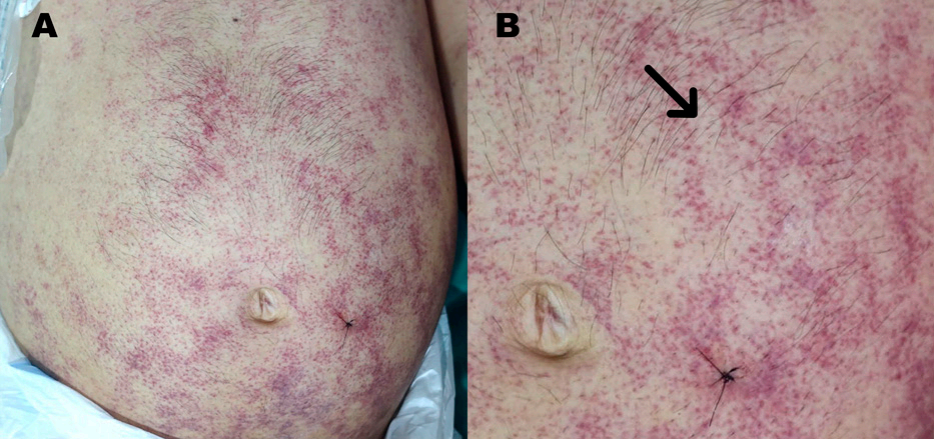
(**A**) Purpuric rash involving the periumbilical area, abdomen, and flank in an HIV patient with disseminated strongyloidiasis. (**B**) Close-up of the rash showing purple macules and papules in the periumbilical area resembling thumbprints (arrow). This figure appears in color at www.ajtmh.org.

During the initial 24 hours, despite fluid resuscitation, the patient deteriorated and required vasoactive drugs and ventilatory support in the intensive care unit, where corticosteroids and sulfamethoxazole/trimethoprim were started for presumed *Pneumocystis* pneumonitis. During intensive care unit admission, a detailed examination of the skin revealed purplish macules and papules on the periumbilical area, abdomen, and flanks (Figure 1A), with linear and serpiginous configurations resembling thumbprints (Figure 1B).^1^^,^^2^

Treatment with oral ivermectin for strongyloidiasis and meropenem as empirical therapy for enteric Gram-negative pathogens^3^ was started; however, despite intensive care and hemodynamic support, the patient worsened progressively to multiple-organ dysfunction and died 6 days after admission.

**Figure 2.**
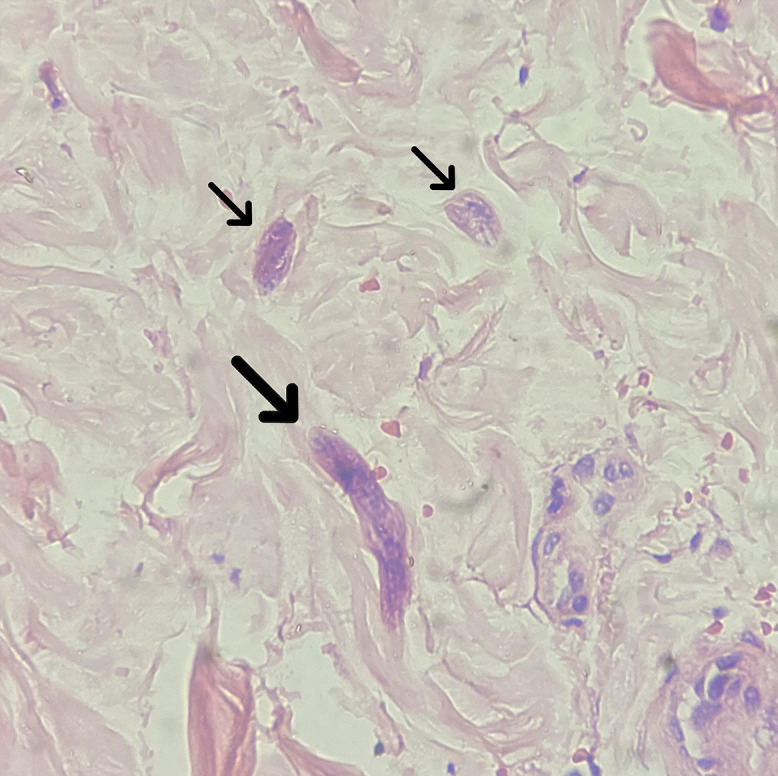
Skin biopsy with filariform of *Strongyloides stercoralis* larva in dermis, full length (thick arrow) and transverse section (thin arrows), surrounded by collagen bundles (hematoxylin–eosin stain, ×40 magnification). This figure appears in color at www.ajtmh.org.

A punch skin biopsy was obtained before the outcome and histological examination showed filariform larvae of *Strongyloides stercoralis* in the dermis, between collagen bundles, with no surrounding inflammatory reaction (Figure 2). The finding of *S. stercoralis* parasites outside the digestive and respiratory tract is the hallmark of disseminated strongyloidiasis.^3^^–^^5^

Disseminated strongyloidiasis is far more associated with human T-cell leukemia virus type 1 co-infection than with HIV infection alone, but unfortunately it could not be ruled out because of the lack of diagnostic testing availability. This clinical picture emphasizes that even in endemic areas for strongyloidiasis, clinical suspicion remains low, delaying treatment and increasing mortality.
